# Frequency-dependent mitochondrial Ca^2+^ accumulation regulates ATP synthesis in pancreatic β cells

**DOI:** 10.1007/s00424-012-1177-9

**Published:** 2012-11-14

**Authors:** Andrei I. Tarasov, Francesca Semplici, Daliang Li, Rosario Rizzuto, Magalie A. Ravier, Patrick Gilon, Guy A. Rutter

**Affiliations:** 1 Section of Cell Biology, Division of Diabetes Endocrinology and Metabolism, Department of Medicine, Imperial College London, SW7 2AZ London, UK; 2 Department of Cell Biology and of Biochemistry, University of Texas Southwestern Medical Center, Dallas, TX 75390-9039 USA; 3 Department of Biomedical Sciences, University of Padua, 35121 Padua, Italy; 4 Institut de Génomique Fonctionnelle, INSERM U661, CNRS UMR5203, Université Montpellier I et II, 34094 Montpellier Cedex 5, France; 5 diabète et nutrition, Pôle d’endocrinologie, Institut de recherche expérimentale et clinique, Université catholique de Louvain, Brussels, Belgium

**Keywords:** Insulin secretion, Calcium, Oscillation, Mitochondria, MCU, ATP

## Abstract

Pancreatic β cells respond to increases in glucose concentration with enhanced metabolism, the closure of ATP-sensitive K^+^ channels and electrical spiking. The latter results in oscillatory Ca^2+^ influx through voltage-gated Ca^2+^ channels and the activation of insulin release. The relationship between changes in cytosolic and mitochondrial free calcium concentration ([Ca^2+^]_cyt_ and [Ca^2+^]_mit_, respectively) during these cycles is poorly understood. Importantly, the activation of Ca^2+^-sensitive intramitochondrial dehydrogenases, occurring alongside the stimulation of ATP consumption required for Ca^2+^ pumping and other processes, may exert complex effects on cytosolic ATP/ADP ratios and hence insulin secretion. To explore the relationship between these parameters in single primary β cells, we have deployed cytosolic (Fura red, Indo1) or green fluorescent protein-based recombinant-targeted (*Pericam*, 2mt8RP for mitochondria; D4ER for the ER) probes for Ca^2+^ and cytosolic ATP/ADP (*Perceval*) alongside patch-clamp electrophysiology. We demonstrate that: (1) blockade of mitochondrial Ca^2+^ uptake by shRNA-mediated silencing of the uniporter MCU attenuates glucose- and essentially blocks tolbutamide-stimulated, insulin secretion; (2) during electrical stimulation, mitochondria decode cytosolic Ca^2+^ oscillation frequency as stable increases in [Ca^2+^]_mit_ and cytosolic ATP/ADP; (3) mitochondrial Ca^2+^ uptake rates remained constant between individual spikes, arguing against activity-dependent regulation (“plasticity”) and (4) the relationship between [Ca^2+^]_cyt_ and [Ca^2+^]_mit_ is essentially unaffected by changes in endoplasmic reticulum Ca^2+^ ([Ca^2+^]_ER_). Our findings thus highlight new aspects of Ca^2+^ signalling in β cells of relevance to the actions of both glucose and sulphonylureas.

**Electronic supplementary material:**

The online version of this article (doi:10.1007/s00424-012-1177-9) contains supplementary material, which is available to authorized users.

## Introduction

A wide range of extracellular stimuli, including receptor agonists and nutrients, trigger cytosolic Ca^2+^ oscillations as a means of transmitting a signal to the cell interior [7]. These Ca^2+^ rises are decoded by calcium binding proteins which ultimately regulate key functions such as energy metabolism [1, 12], growth and development [54], activation [15] and apoptosis [37]. Pulsatile Ca^2+^ changes are thus important both (a) to provide the opportunity for intricate spatiotemporal control of intracellular events [48] and (b) to avoid the need for a sustained calcium rise, which may provoke undesirable effects such as the formation of insoluble calcium complexes and potentially catastrophic structural damage. At present, however, the machinery which distinguishes pulsatile Ca^2+^ rises from a sustained increase of the same amplitude is only partly defined [15].

Glucose induces the secretion of insulin from the pancreatic β cell through the stimulation of oxidative metabolism, an elevation in cytosolic ATP/ADP ratios and the closure of ATP-sensitive K^+^ channels (K_ATP_). The subsequent depolarisation of the plasma membrane then leads to bursting electrical activity, the influx of Ca^2+^ through voltage-gated Ca^2+^ channels and the activation of secretory granule exocytosis [44, 55]. In addition, further mechanisms, independent of K_ATP_ channels, amplify the effects of Ca^2+^ [23]. Mitochondrial metabolism is particularly important in the triggering of insulin secretion [30], with >95 % of glucose-derived carbon atoms being oxidised within these organelles [50, 55] in a process further stimulated by activation of the glycerol phosphate shunt by [Ca^2+^]_cyt_ [45]. Increases in mitochondrial free calcium ([Ca^2+^]_mit_) in response to glucose [1, 25] or other stimuli [47] have also been suggested to activate intramitochondrial dehydrogenases [12, 42]. These events are together likely to stimulate mitochondrial ATP synthesis, providing a positive feedback mechanism to further enhance insulin secretion [43].

We have recently developed an approach to monitoring intracellular free Ca^2+^ in multiple compartments simultaneously in the same living cell, whilst measuring (or manipulating) membrane potential through the patch pipette [51, 53]. Thus, the mitochondrial matrix-targeted Ca^2+^ sensitive probe, *pericam*, is used alongside an intracellularly trappable calcium probe, Fura red [52], to monitor changes in mitochondrial and cytosolic free Ca^2+^, respectively [51]. Likewise, the ATP-sensitive probe *Perceval* [6] can be used to monitor changes in cytosolic ATP/ADP ratio simultaneously with cytosolic Ca^2+^ [51]. In this way, we have demonstrated [53] that glucose-induced increases in intramitochondrial free Ca^2+^, mediated by the mitochondrial uniporter (MCU) [5, 11] and modulated by the Na^+^–Ca^2+^ exchanger NCLX, are required for normal increases in cytosolic ATP/ADP ratio. On the other hand, neither the relationship between [Ca^2+^]_cyt_ and [Ca^2+^]_mit_ during temporally complex changes in the former nor the relationship between cytosolic Ca^2+^ and Ca^2+^ in other organelles, e.g. the endoplasmic reticulum, was explored.

An interesting finding made during our earlier studies [53] was that increases and decreases in intramitochondrial Ca^2+^ are significantly delayed with respect to those in the cytosol when these are changed in a single step. This has raised the question as to how mitochondrial Ca^2+^ may respond to repetitive, oscillatory changes in cytosolic Ca^2+^. This issue is of particular physiological importance since both glucose-induced cytosolic Ca^2+^ increases [47] and insulin secretion [34] are pulsatile in nature, a feature that may be driven by glycolytic oscillations [8] and the complex interplay of ion channel activities [32] which lead to bursts in electrical activity. Whether oscillatory changes in mitochondrial Ca^2+^, and hence pulses in ATP synthesis, can then occur and may contribute to the control of insulin secretion has yet to be determined [2, 13].

We therefore sought here to determine how oscillations in electrical activity, and hence cytosolic Ca^2+^, are decoded by β cell mitochondria in situ. We also examined the impact of cytosolic [Ca^2+^] oscillations on the concentration of this ion within the endoplasmic reticulum and assessed whether fluxes between the Ca^2+^ and the ER may influence [Ca^2+^]_mit_ dynamics.

We show that attenuation of [Ca^2+^]_mit_ increases, achieved by silencing MCU, inhibits insulin secretion, demonstrating the importance of mitochondrial calcium accumulation for the normal regulation of hormone release from primary β cells. We then demonstrate that the *amplitude* of the [Ca^2+^]_mit_ increases displays a remarkable dependence upon the *frequency* of [Ca^2+^]_cyt_ oscillations. Thus, “frequency-amplitude decoding” of the oscillations by mitochondria allows the modulation of ATP/ADP production whilst bypassing the need for stable, and potentially damaging, increases in [Ca^2+^]_cyt_.

## Materials and methods

### Islet isolation and culture

Female CD1 mice (10–12 weeks of age) were obtained from Charles Rivers (Margate, UK) and fed ad libitum prior to use. After cervical dislocation according to UK Home Office approved procedures (Animals Scientific Procedures Act, 1986), pancreatic islets were isolated by infusion of collagenase via the pancreatic duct [41]. After pre-culture for 5 h in RMPI-1640 medium, containing 11 mM glucose, 10 % FCS, 100 μU penicillin, 100 μg streptomycin, at 37 °C, 5 % CO_2_, in absolute humidity, islets were infected with adenoviruses delivering the cDNA encoding the required probe, split into single β cells by mechanical disruption and plated on glass coverslips. Cells were then cultured for a further >24 h for 2–5 days and assayed as described below. Glass-attached single cells or two- to three-cell clusters displayed an infection efficiency of >90 % [41].

### Molecular biology and generation of adenoviruses

Adenoviruses encoding the ATP/ADP sensor Perceval [6] or the mitochondrial Ca^2+^ sensor 2mt8-ratiometric pericam (2mt8RP) [18] were generated as described [51]. MCU was silenced using a suitable lentivirus (Santa Cruz) as described [53] and verified by qRT-PCR.

To measure [Ca^2+^]_ER_, an adenovirus encoding D4ER was used [40]. This construct includes cDNA encoding D1ER [35], modified by replacing the Ca^2+^ binding domain D1 with the low affinity Ca^2+^ sensor D4, downstream of the rat insulin promoter. Recombination with pAdEasy-1, transfection into HEK293 cells and adenoviral particle production were achieved according to published protocols [28].

### Single cell epifluorescence imaging

Simultaneous imaging of free [Ca^2+^] in mitochondria and in the cytosol was performed essentially as described [51] using the mitochondrial pericam 2mt8RP and Fura-Red (Invitrogen), respectively; [Ca^2+^] was measured simultaneously in the ER and cytosol using D4ER cameleon and Indo-1 (Invitrogen). 2mt8RP, Fura-Red and Indo-1 were examined at single excitation and emission wavelengths. Either dye was dissolved in DMSO (4 mM) containing 4 % F127-Pluronic. Cells were loaded by incubation with either dye (4 μM) in the extracellular solution for 30 min. Imaging experiments were performed on an Olympus IX-71 microscope with a UPlanFL N × 40, NA 1.2 objective. For acquisition, an F-View-II camera and MT-20 excitation system equipped with a Hg/Xe arc lamp were used, under control of Cell^R software (Olympus). The excitation/emission wavelengths were (nanometres): 410 of 535 (2mt8RP), 490 of 630 (Fura-Red), 490 of 535 (perceval), 440 of (465 and 530) (D4ER cameleon) and 350 of 465 (Indo-1). In all cases, cells were maintained at 3 mM glucose for 15–30 min prior to stimulation at the indicated glucose concentrations. Images were acquired at a frequency of 0.2 Hz (apart from Fig. 2a, where a frequency of 2 Hz was used). Every Ca^2+^ recording was subjected to the dynamic range control by applying, at the end of the trace, solutions containing 10 μM ionomycin: “Ca^2+^-free” (0.5 mM EGTA) and “Ca^2+^-max” (5 mM Ca^2+^). The acquisition of the fluorescence and electrophysiological data was synchronized using TTL pulse.

Imaging data were background-subtracted, analysed and presented as *F*/*F*_0_ (perceval), *R*/*R*_0_ 530/465 (D4ER) and *F*_0_/*F* (Fura-Red, Indo-1, 2mt8RP). Whole cells were selected as regions of interest (ROI) to minimize the drift effects during the long recordings. In the case of a cell cluster, only the cell that was patched was included in the ROI. Changes in the fluorescence of zinc indicator for measuring induced exocytosis of zinc (ZIMIR) were measured as previously described [26]. The properties of the FRET probes used here are described in previous publications [6, 40, 53].

### Electrophysiology

Electrophysiological recordings and stimulation were done in the whole-cell perforated-patch configuration, using an EPC9 patch-clamp amplifier controlled by Pulse acquisition software (HEKA Elektronik). The pipette tip was dipped into pipette solution and then back-filled with the same solution containing 0.17 μg/ml amphotericin B. Series resistance and cell capacitance were compensated automatically by the acquisition software. Recordings, triggered by the TTL pulse, were started in current-clamp mode, and the depolarization of the plasma membrane was monitored simultaneously with [Ca^2+^] and [ATP/ADP]_cyt_, in response to a glucose step from 3 to 10 or 16.7 mM. After the onset of electrical activity, the mode was switched to voltage clamp to control *V*_m_ and impose electrical stimulations. *V*_m_ was held at the value of −70 mV. Electrical stimulation was imposed to mimic the naturally occurring bursts of action potentials and comprised 5-s depolarization trains to −30 mV containing 25 ramps of 100 + 100 ms to 0 mV and back (Suppl. Fig. S1). The artificial bursts were applied at different frequencies: every 10, 4, 2, 1 and 0.5 min^−1^, which correspond to “duty cycle” values of 0.9, 0.36, 0.18, 0.09 and 0.045, respectively. Data were filtered at 1 kHz and digitised at 2 kHz.

### Experimental solutions

The pipette solution contained (millimolar): 76 K_2_SO_4_, 10 NaCl, 10 KCl, 1 MgCl_2_, 5 HEPES (pH 7.35 with KOH). The extracellular bath solution, referred in text as “EC” contained (millimolar): 120 NaCl, 4.8 KCl, 24 NaHCO_3_ (saturated with CO_2_), 5 HEPES (pH 7.4 with NaOH), 2.5 CaCl_2_, 1.2 MgCl_2_. All experiments were conducted at 32–33 °C, and the bath solution was perifused continuously.

### Data analysis

Imaging data were analysed using Cell^R (Olympus), ImageJ (Wayne Rasband, NIMH) and MS Excel. Simultaneous recordings were combined and analysed using Igor Pro (Wavemetrics). The results are presented as mean ± SEM. A Mann–Whitney *U* test was used to assess the statistical significance of the differences between the independent samples. Wilcoxon’s paired test was used in case of dependent samples. The significance values are specified in each figure; as a rule, *p* < 0.05 differences were considered significant.

## Results

### Mitochondrial Ca^2+^ accumulation mediated by MCU modulates insulin secretion from primary mouse β cells

Having recently reported that Ca^2+^ uptake by mitochondria is essential for glucose-stimulated ATP increases in pancreatic β cells [53], we sought firstly to determine whether MCU-mediated mitochondrial Ca^2+^ uptake was required for the stimulation of insulin secretion in this system. To this end, we used monolayer cultures of dispersed mouse islets, comprising single cells or small clusters of two to three cells [41]. In this preparation, MCU expression could readily be decreased by ∼80 % through lentivirus-mediated delivery of a short hairpin RNA (shRNA; see “Materials and methods”) [53].

To assay insulin secretion from *single* cells, we used a fluorescent cell surface-attached dye, ZIMIR, which detects Zn^2+^ co-secreted with insulin [26]. In this assay, changes in ZIMIR fluorescence reflect the balance between the release of Zn^2+^ from the β cells and chelation of the released Zn^2+^ by low concentrations of EGTA, present in extracellular buffer. Thus, the ZIMIR signal indicates the *rate* of secretion rather than the absolute amount of secreted insulin.

**Fig. 1:**
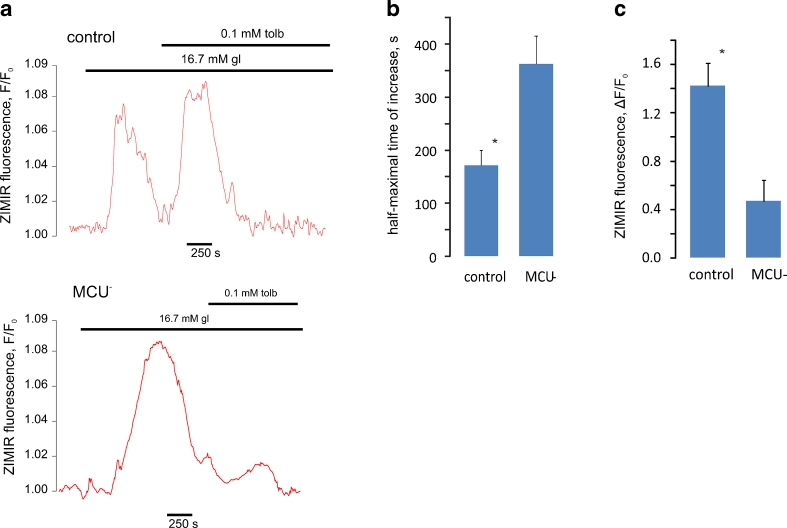
The mitochondrial Ca^2+^ uniporter MCU is required for the stimulation of insulin secretion from primary β cells by glucose and sulphonylureas. **a** As a surrogate for insulin secretion, the release of Zn^2+^ was imaged using ZIMIR in single β cells. The cells were infected with lentivirus encoding for non-sense or anti-MCU shRNA, as indicated. Cells were exposed to high glucose as shown, after which the sulphonylurea tolbutamide was added. **b** Half-maximal times of the increase in ZIMIR fluorescence induced by 16.7 mM glucose. **c** Effect of MCU silencing on the amplitude of tolbutamide-induced increases in ZIMIR fluorescence. *P < 0.05, differences are statistically significant

Silencing of MCU led to a delay in glucose-induced ZIMIR increases (Fig. 1a–c), consistent with an inhibition of insulin secretion [26], although the amplitude of the final increase was unchanged in MCU-depleted cells. Strikingly, the effects of further stimulation with the sulphonylurea tolbutamide were substantially decreased in MCU-depleted cells (Fig. 1a, c). Sulphonylureas, which are first-choice drugs for treatment of type 2 diabetes mellitus, specifically inhibit β cell K_ATP_ channels, depolarise the plasma membrane and induce electrical activity even at low glucose [4]. At high glucose, when the β cell is already electrically active, addition of sulphonylurea is known to result in a substantial increase in the frequency of electrical spiking [17] (and data not shown). The above data (Fig. 1a, c) thus suggest that the ability of this additional excitation to stimulate exocytosis relies upon Ca^2+^ entry into mitochondria. This prompted us to explore how electrical spikes, inducing cytosolic Ca^2+^ increases, are linked to Ca^2+^ increases in mitochondria and to the consequent increase in ATP generation by these organelles.

### Dynamics of β cell [Ca^2+^]_cyt_ and [Ca^2+^]_mit_ during glucose-induced electrical activity

**Fig. 2:**
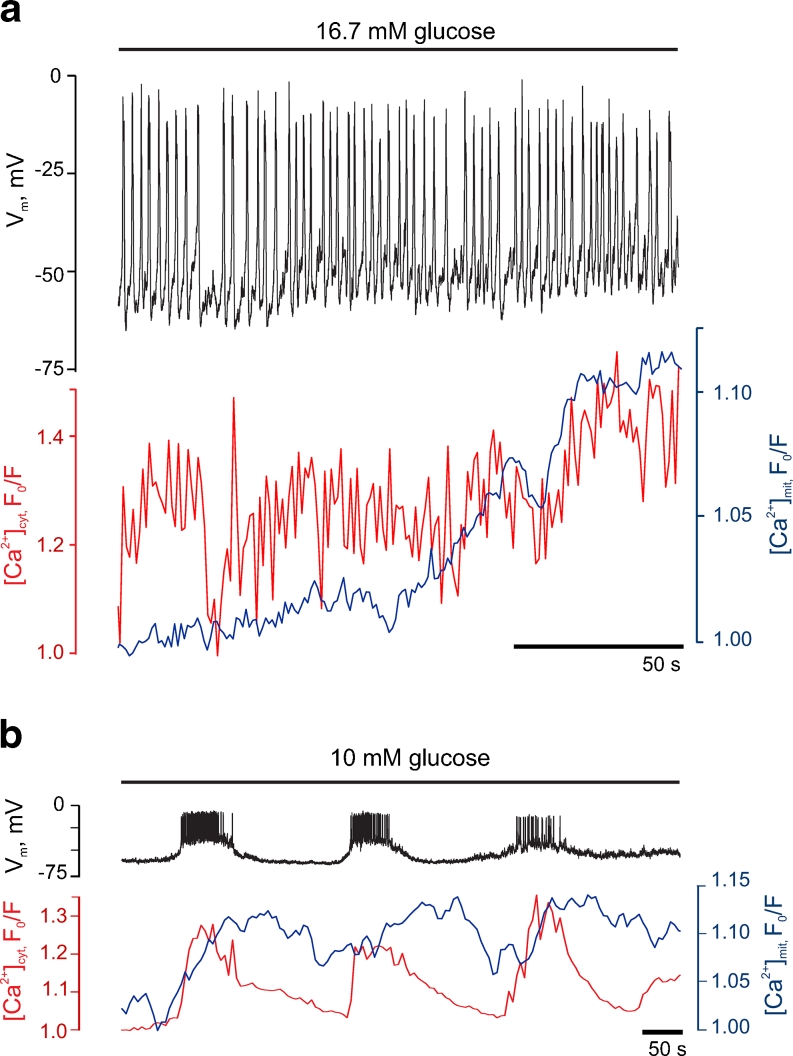
Glucose-induced cytosolic Ca^2+^ oscillations are followed by damped increases in mitochondrial free Ca^2+^ in primary β cells. **a** Regenerative electrical activity (*black*) at 16.7 mM glucose and corresponding changes in [Ca^2+^]_cyt_ (*red*) and [Ca^2+^]_mit_ (*blue*). **b** Representative trace of changes in electrical activity (*upper trace*, *black*), [Ca^2+^]_cyt_ (*red*) and [Ca^2+^]_mit_ (*blue*) induced by 10 mM glucose in a separate single β cell. The results are representative of at least ten further trials from different preparations

In cells in which electrical activity was stimulated by the presence of 16.7 mM glucose, Fura-Red and 2mt8RP were used to report the Ca^2+^ signals from the cytosol and mitochondrial lumen, respectively, whilst plasma membrane potential (*V*_m_) was measured simultaneously using the perforated patch configuration. Remarkably, the majority of individual electrical spikes prompted by glucose at 1.1 ± 0.1Hz (*n* = 37 cells) were reliably tracked by increases and decreases in [Ca^2+^]_cyt_ whilst [Ca^2+^]_mit_ was increased only after a much longer lag (Fig. 2a). Indeed, increases in [Ca^2+^]_mit_ were difficult the resolve at each peak (Fig. 2a) though the progressive elevation of [Ca^2+^]_mit_ observed suggested that they may occur, but reverse slowly.

We next asked how [Ca^2+^]_mit_ might respond to more widely spaced oscillations in electrical activity and [Ca^2+^]_cyt_, induced by an intermediate concentration of glucose. At 10 mM glucose, *V*_m_ typically displayed periodic bursting activity [33], with frequencies of one burst every 3–4 min (215 ± 33 s; *n* = 6 cells from three separate preparations; Fig. 2b). The onset of each burst was quickly followed by an elevation in [Ca^2+^]_cyt_, reaching a maximum value in 10–20 s, whilst [Ca^2+^]_mit_ increased more slowly, taking ∼1 min to plateau. The termination of each electrical burst was accompanied by a rapid relaxation of [Ca^2+^]_cyt_ to basal levels whilst [Ca^2+^]_mit_ remained significantly elevated for 1–2 min, barely falling before the onset of the next burst (Fig. 2b). Thus, mitochondria discriminate between continuous spiking (every 1.5 s) and slow bursting (every 215 s) induced by glucose. In the former case, mitochondria display continuously elevated Ca^2+^ whilst in the latter case [Ca^2+^]_mit_ tracks changes in *V*_m_ and [Ca^2+^]_cyt_.

### The amplitude of [Ca^2+^]_mit_ increases depends on the frequency of electrically imposed [Ca^2+^]_cyt_ oscillations

The above observations suggested that the degree of Ca^2+^ accumulation by mitochondria, and thus the *amplitude* of the observed [Ca^2+^]_mit_ increases, may depend on the *frequency* of *V*_m_ bursts and hence [Ca^2+^]_cyt_ increases. Thus, lower frequency pulses in [Ca^2+^]_cyt_ might be expected to allow [Ca^2+^]_mit_ to return to basal levels before the next spike, whereas higher-frequency pulses might allow the accumulation of Ca^2+^ by mitochondria and hence progressive increases in [Ca^2+^]_mit_ (“summation”).

In practice, [Ca^2+^]_cyt_ changes of a specific frequency cannot readily (i.e. reproducibly between different single β cells) be imposed through the modulation of glucose concentrations alone. Moreover, step increases in glucose concentration would in any case be expected to enhance glycolytic and oxidative metabolism though a substrate supply effect. This, in turn, should increase respiratory chain activity and mitochondrial ATP synthesis independently of any action of Ca^2+^ on intramitochondrial metabolism. To avoid these confounding effects, we therefore imposed [Ca^2+^]_cyt_ oscillations of varying frequency by manipulating the plasma membrane potential, and hence the activity of voltage-gated Ca^2+^ channels, using voltage clamp.

**Fig. 3:**
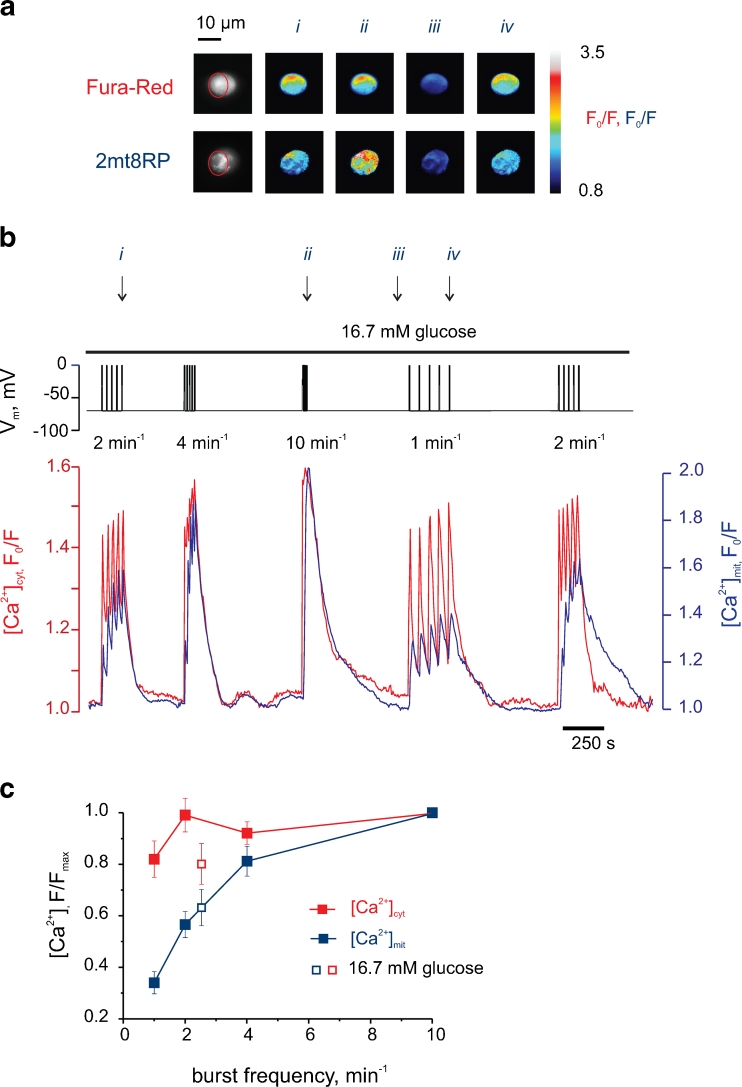
The amplitude of [Ca^2+^]_mit_ increases is regulated by the frequency of electrical bursts. **a** Epifluorescent images of the cell analysed in **B** with the ROI indicated (*red*) and pseudo-colour pixel-to-pixel ratios measured at four sequential time points. **b** Simultaneous recording of [Ca^2+^]_cyt_ (*red*), [Ca^2+^]_mit_ (blue) and *V*_m_ (*upper trace, black*). The cell was bathed in EC containing 16.7 mM glucose, with *V*_m_ being held at −70 mV. The depolarisations were then imposed using the voltage clamp, with different rates being applied in random order. The arrows (*i–iv*) indicate the time points corresponding to the cell images in Fig. 3a. **c** Effect of the bursting frequency on the [Ca^2+^]_cyt_ (*solid red*) and [Ca^2+^]_mit_ (*solid blue*), *n = 13*. Maximal values of [Ca^2+^]_cyt_ and [Ca^2+^]_mit_ of each five-burst train were normalised to the maximal values of the trains with maximal frequency (10 min^−1^), measured in the same cell. The [Ca^2+^]_cyt_ and [Ca^2+^]_mit_ data points that correspond to naturally occurring electrical activity (at 16.7 mM glucose, measured in the current clamp on the same cell) are given as *open red* (*n = 7*) and *open blue* (*n* = 7) *squares*, respectively. The differences between the neighbour points of [Ca^2+^]_mit_ curve are statistically significant (*P* < 0.02)

We thus applied depolarisations of constant amplitude (Fig. S1A) but varying frequency (1–10 min^−1^; Fig. 3a, b). The parameters of the stimulation protocol were chosen to mimic the electrical activity that occurs naturally in intact islets [20]. Examined at 16.7 mM glucose, stimulation at each of the frequencies tested caused clear and large increases in [Ca^2+^]_cyt_, reflecting influx across the plasma membrane (Fig. 3a, b). Indeed, the amplitude of the [Ca^2+^]_cyt_ increases was essentially maximal even at the lowest frequency tested (1.0 min^−1^; Fig. 3b). By contrast, the amplitude of the corresponding [Ca^2+^]_mit_ peaks increased progressively with depolarisation frequency, effectively doubling between 1 and 2 min^−1^ and again between 2 and 10 min^−1^ (Fig. 3b). The relationship between burst frequency, and the mean amplitude of the [Ca^2+^]_cyt_ and [Ca^2+^]_mit_ changes, is shown in Fig. 3c. This analysis revealed a marked right-shift in the response to burst frequency of [Ca^2+^]_mit_ when compared to [Ca^2+^]_cyt_. For example, in the case of [Ca^2+^]_mit_, ∼4 bursts min^−1^ were required to achieve 80 % *F*/*F*_max_. By contrast, the same *F*/*F*_max_ was obtained for [Ca^2+^]_cyt_. with a burst frequency of just ≤1 min^−1^.

In order to confirm the physiological validity of the stimulation protocol chosen above, we also monitored the values of [Ca^2±^]_cyt_, [Ca^2±^]_mit_ and *V*_m_ during the application of natural stimulus, high glucose (16.7 mM), alone (Fig. S1B). Subsequent voltage clamping of the same cell then allowed us to impose depolarisations at frequencies (five bursts at 4 min^−1^ for the cell shown in Fig. S1) which closely replicated the changes of [Ca^2±^]_cyt_ and [Ca^2±^]_mit_ during the prior exposure to elevated glucose. Importantly, the frequency of bursts required to mimic stimulation with glucose typically lay mid-range in the plot of burst frequency vs. [Ca^2±^]_cyt_ and [Ca^2±^]_mit_ shown in Fig. 3c (open squares).

Similar data to those above were obtained at 10 mM glucose (Fig. S2), whereas recordings at lower (3 mM) glucose led to a deterioration in the Ca^2+^ changes in both compartments (not shown), consistent with a requirement for sustained elevation in glucose metabolism and ATP synthesis for intracellular Ca^2+^ homeostasis during the above protocols.

### “Summation” of cytosolic Ca^2+^ oscillations by mitochondria

**Fig. 4:**
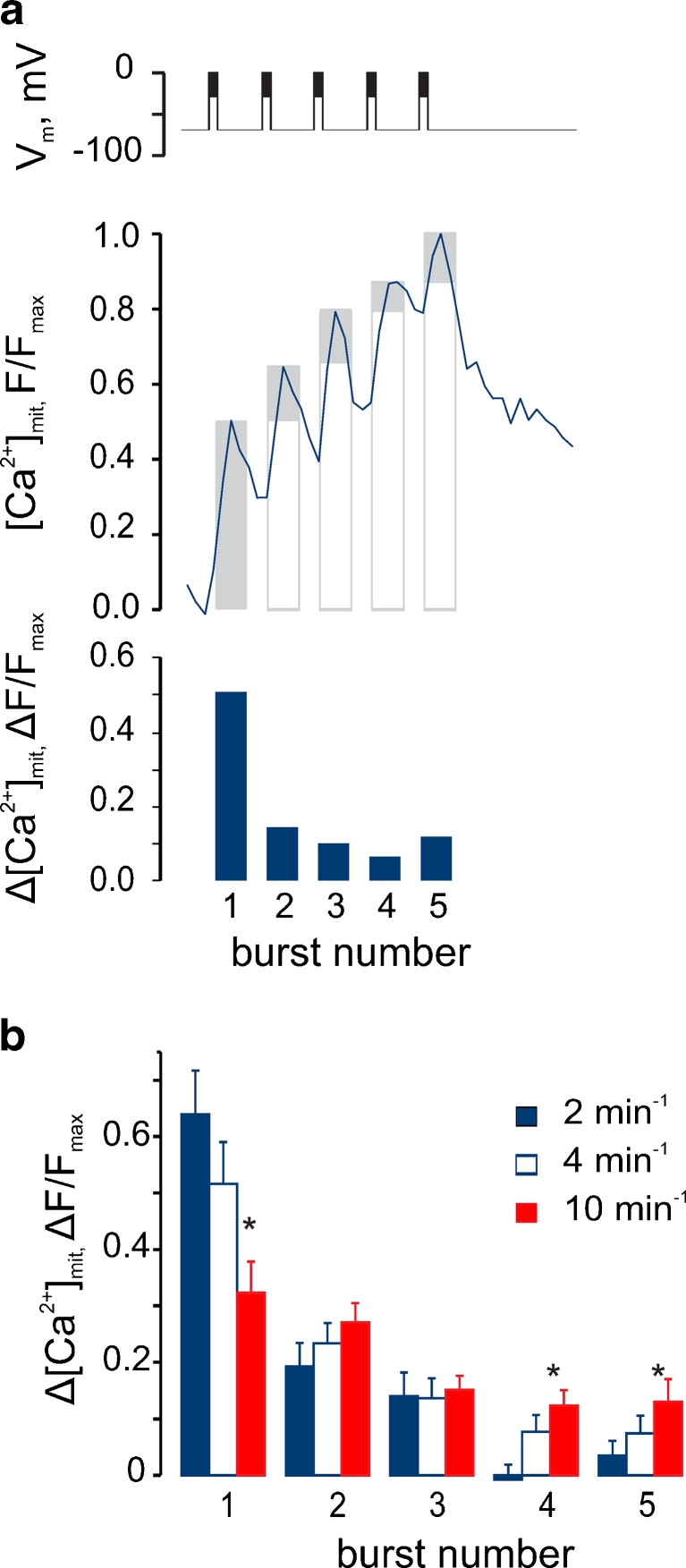
Summation of mitochondrial Ca^2+^ depends on the bursting frequency. **a** Determination of the differential contribution of each burst (∆[Ca^2+^]_mit(*n*)_) for a 2-min^−1^ train. *Upper graph*: the *columns* present maximal [Ca^2+^]_mit_ within the burst normalized to maximal [Ca^2+^]_mit_ within the whole five-burst train ([Ca^2+^]_mit(*n*)_)/[Ca^2+^]_mit(max)_). *Lower graph*: differential contribution of each burst (∆[Ca^2+^]_mit(*n*)_). The *columns* represent the differences between each column and the preceding one, in the *upper graph*. **b** The differential contribution of each burst at different bursting frequencies. *P < 0.05, differences between the data at 2 and 10 min^−1^ are statistically significant

To probe further the properties of the frequency-sensing mechanisms of mitochondria, we analysed in detail the response of [Ca^2+^]_mit_ to stimulation by depolarising trains of different frequencies. The contribution of the each [Ca^2+^]_mit_ peak to the maximum [Ca^2+^]_mit_ amplitude achieved after the firing of five bursts (Fig. 4a) was quantified. This analysis revealed that the fraction of the maximal [Ca^2+^]_mit_ reached after the first burst progressively decreased with burst frequency (Fig. 4b). Thus, at a low burst frequency (2 min^−1^), the increase in [Ca^2+^]_mit_ provoked by the first burst represented ∼70 % of maximum. By contrast, at the highest frequency tested (10 min^−1^), the first burst achieved ∼30 % of the maximum [Ca^2+^]_mit_ increase. The values at intermediate burst frequencies lay between these extremes. Thus, the magnitude of the final [Ca^2+^]_mit_ increase was dependent on the summation of individual [Ca^2+^] increases that failed to relax to the baseline between depolarisation bursts.

### Role of the endoplasmic reticulum in the interplay between [Ca^2+^]_cyt_ and [Ca^2+^]_mit_

**Fig. 5:**
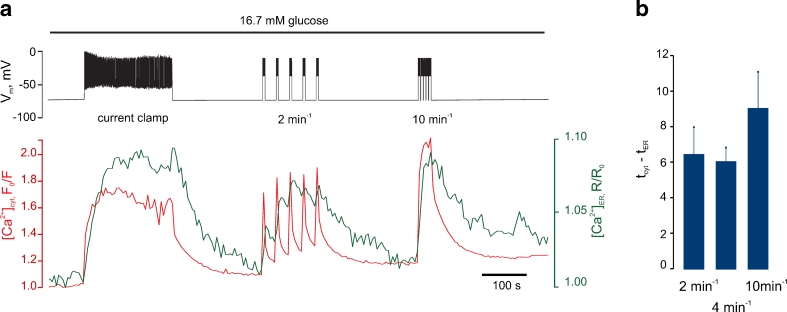
Effect of electrical bursting frequency on the apparent sequestration of Ca^2+^ by the ER. **a** Depolarisation-induced dynamics of [Ca^2+^]_ER_, data are a representative of *n* = 6 traces. The cell was bathed in the EC solution containing 16.7 mM glucose at 37 °C and [Ca^2+^]_ER_ (*green trace*), [Ca^2+^]_cyt_ (*red trace*) and *V*_m_ (upper trace) were monitored/manipulated simultaneously. The cell was held at *V*_m_ = −70 mV initially, then the voltage clamp was released, as indicated by “current clamp” label on the *V*_m_ trace and action potential spiking was observed. The cell was next re-clamped at −70 mV, and after [Ca^2+^]_cyt_ and [Ca^2+^]_ER_ had reached the basal levels, the depolarisation protocol (Suppl. Fig. S1) was applied at the frequency of 4 and 10 min^−1^, as indicated. **b** The delay between maximal increase in [Ca^2+^]_cyt_ and [Ca^2+^]_ER_ in response to five depolarizing trains applied at 2, 4 and 10 min^−1^

We next asked whether the kinetics of the increases in Ca^2+^ in the ER may show a similar dependence on [Ca^2+^]_cyt_ oscillation frequency to those for the mitochondrial changes measured above. ER Ca^2+^ ([Ca^2+^]_ER_) was therefore measured using the probe D4ER [40] (Fig. S3), whilst cytosolic Ca^2+^ was imaged using Indo-1. During the same depolarisation protocols as used above, ER free Ca^2+^ changes were also dependent on pulse frequency (Fig. 5a vs. Fig. 3b, c). However, ER sequestered Ca^2+^ from the cytosol equally efficiently at low (1 min^−1^) and high (10 min^−1^) bursting rates (Fig. 5b). Thus, the summation of [Ca^2+^]_mit_ increases could not be explained by local effects of the non-sequestered cytosolic Ca^2+^.

We next attempted to explore the potential contribution of ER Ca^2+^ uptake and release in sculpting the relationship between [Ca^2+^]_cyt_ and [Ca^2+^]_mit_. To this end, we tested the effects of the sarco-(endo)plasmic reticulum Ca^2+^-ATPase inhibitor cyclopiazonic acid (CPA). As expected, 10 μM CPA abolished apparent Ca^2+^ accumulation into the ER (Fig. S4A) whereas a similar frequency dependence of [Ca^2+^]_mit_ on [Ca^2+^]_cyt_ pulses was observed as in the absence of the inhibitor (Fig. S4B vs. Fig. 3b). However, the use of this inhibitor was complicated by the fact that larger increases in [Ca^2+^]_cyt_ were observed at each pulse, reflecting the functional loss of this important intracellular store (Fig. S4B).

Interestingly, activation of Ca^2+^ influx across the plasma membrane increased [Ca^2+^]_mit_ more efficiently that the mobilisation of intracellular calcium (Fig. S5), in line with previous results [48]. Thus, when the former was activated by exposure to stepped pulses in extracellular K^+^ concentration, or the latter by exposure to varying acetyl choline concentrations (Fig. S5A), then for a given increase in [Ca^2+^]_cyt_, the increase in [Ca^2+^]_mit_ was larger after the activation of Ca^2+^ influx from the extracellular space (Fig. S5B).

### Cytosolic ATP/ADP increases are controlled by electrical bursting and [Ca^2+^]_cyt_ oscillation frequency

**Fig. 6:**
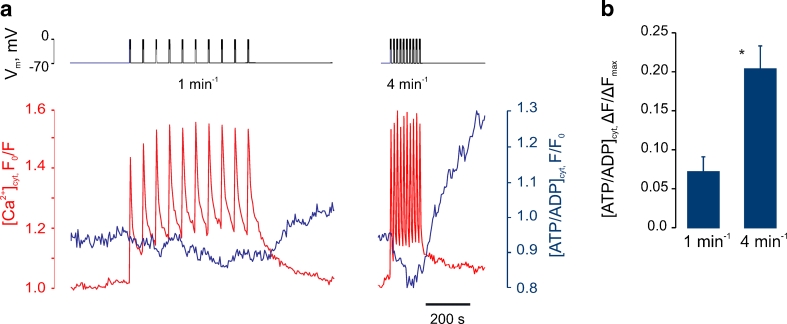
Electrical bursting frequency controls cytosolic ATP/ADP increases. **a** β Cells were voltage-clamped at −70 mV at 16.7 mM glucose and 10-burst trains of depolarisations were imposed at 1 or 4 min^−1^. [ATP/ADP]_cyt_ and [Ca^2+^]_cyt_ were reported by Perceval and Fura-Red, respectively. **b** Mean [ATP/ADP]_cyt_ potentiation in response to depolarisation at 1 min^−1^ (*n =* 14) or 4 min^−1^ (*n* = 16). The data were normalised to the width of the range of [ATP/ADP]_cyt_ change between application of 16.7 mM glucose and 2 μM FCCP (Δ*F*_max_). *P < 0.01, differences are significant

We sought next to explore the functional consequences of the above relationship between [Ca^2+^]_cyt_ and [Ca^2+^]_mit_ (Fig. 3b, c) by monitoring the cytosolic ATP/ADP ratio ([ATP/ADP]_cyt_) in real time with the recombinant green fluorescent protein-based probe *perceval* [6]. Extending our recent observations [51], we observed that the imposition with the patch pipette of [Ca^2+^]_cyt_ oscillations at low frequency (1.0 min^−1^) had no discernible effect on the basal [ATP/ADP]_cyt_ (Fig. 6a), consistent with the small changes in [Ca^2+^]_mit_ which occur during this protocol (Fig. 3b, c). By contrast, increasing the frequency of bursts to 4 min^−1^ caused first a transient decrease in [ATP/ADP]_cyt_ and then a progressive increase in this ratio which, remarkably, continued after the termination of the pulses. Thus, the maximal amplitude of the [ATP/ADP]_cyt_ increases induced by bursts imposed at 4 min^−1^ was 2.8 ± 1.2 times higher than that induced by a pulse rate of 1 min^−1^ (Fig. 6b).

## Discussion

The principal aims of the present study were to assess the role and regulation of mitochondrial Ca^2+^ uptake and ATP synthesis during physiological (i.e. pulsatile) changes in cytosolic Ca^2+^. To this end, we deployed our recently developed system [51] for imaging multiple parameters simultaneously in single cells, using the patch pipette to record or non-invasively manipulate plasma membrane potential and hence cytosolic Ca^2+^ with high precision. This approach, involving the use of a dispersed islet preparation, mimics many aspects of electrical and cytosolic Ca^2±^ oscillations observed for cells in situ within intact islets [49]. Although local interactions between small numbers of β cells within a cluster are retained, the preparation may not, however, fully replicate the collective effects of interactions across large number of β cells in the whole islet. Nevertheless, for studies of the sort undertaken here, this preparation provides marked advantages. Firstly, the expression of genes can be readily achieved and manipulated by viral gene (or shRNA) delivery, whereas the poor penetration of viral particles into the intact islet [14] limits the use of the latter for such studies. Secondly, during imaging, interference with signals from the recombinant FRET probes by endogenous autofluorescence is markedly reduced compared to intact islets [41] (not shown). Lastly, the use of individual cells and clusters greatly facilitates the manipulation of the membrane potential through the patch pipette.

We show that silencing of *ccdc109a* (MCU) [5, 11], recently suggested to mediate Ca^2+^ entry into mitochondria as a complex with the regulator subunit MICU1 [36], inhibits glucose-induced insulin secretion to a small extent, whilst essentially eliminating secretion stimulated by tolbutamide (Fig. 1a–c). Since the effects of sulphonylureas are mediated by the elevation of the frequency of electrical spiking [17], we went on to examine in detail how individual electrical spikes are decoded by β cell mitochondria. We found that, under physiological conditions, changes in [Ca^2+^]_mit_ in β cells are governed by the frequency of electrical bursting. Together with our earlier findings [1, 38, 51], our present results indicate the existence of a causal link between electrical activity and Ca^2+^-mediated increases in mitochondrial ATP production.

We would stress that here, and in our earlier studies [40, 53], precise calibration of [Ca^2+^] signals could not routinely be achieved. Nonetheless, post-experiment treatment with ionomycin, in high Ca^2+^-containing solutions, was used to determine whether the probes were likely to have reached saturation. From these trials, and previous calibration of the mitochondrially targeted probe [19], which has indicated a *K*_d_ value of 1 μM in situ, we estimate that maximal [Ca^2+^]_mit_ levels extended into the low micromolar range at during peaks [47], exceeding those of [Ca^2+^]_cyt_ (<1 μM).

### Role of mitochondrial Ca^2+^ transport mediated by MCU in the control of insulin secretion

We have recently shown that silencing of the mitochondrial uniporter MCU has no detectable effect on the first phase (within ∼5 min) of glucose-induced electrical activity or ATP increase [51] whereas the second phase of glucose-induced ATP/ADP increase was sharply reduced. In the present study, we report that silencing of MCU leads to a modest inhibition of glucose-induced secretion of insulin, assayed with single-cell resolution by measuring the release of Zn^2+^ with the membrane-bound probe ZIMIR [26]. However, the further stimulation of insulin exocytosis by sulphonylureas was almost completely abolished.

The use of ZIMIR [26] in the present studies allowed a convenient means to study insulin secretion at the level of single cells and thus comparison with our subsequent measurements of Ca^2+^ and ATP dynamics in this preparation. However, this approach requires the continuous chelation of the secreted Zn^2+^. The probe therefore allows a semi-quantitative assessment of the *rate* of insulin release, but is not optimal for dissecting the contributions of the first and second phases of secretion. Nonetheless, we were able to demonstrate effects on both glucose-and tolbutamide-stimulated secretion with this tool. Strikingly, the effects of tolbutamide were markedly (by >80 %) diminished in MCU-silenced cells (Fig. 1a, c). We suspect that this may reflect diminished secretory granule competence (or translocation towards release sites), perhaps as a result of impaired mitochondrial ATP synthesis. In support of this view, our earlier studies [53] revealed a marked decrease in the second phase of glucose-induced ATP increase in MCU-silenced vs. scrambled shRNA-treated cells. Thus, at the time point of addition of tolbutamide in the experiments performed here (≥600 s after the addition of high glucose; Fig. 1), the cytosolic ATP/ADP ratio in MCU-silenced cells is expected to be significantly lower than in control cells.

A recent report has shown that MCU silencing impairs insulin secretion stimulated from clonal rat insulinoma cells, INS-1(832/13), by 16 mM glucose [3]. Although clonal β cells display a number of metabolic and secretory abnormalities (see [50]), a ∼50 % decrease in insulin secreted within 1 h [3] agrees well with the idea that the second phase of insulin secretion relies upon mitochondrial Ca^2+^ entry. This interpretation is, furthermore, consistent with the selective impairment of the second phase of glucose-stimulated insulin secretion from rat islets in which mitochondrial Ca^2+^ increases were buffered with the Ca^2+^ binding protein, S100G [55].

### Frequency control of mitochondrial Ca^2+^ accumulation and ATP synthesis

Given the importance of mitochondrial Ca^2+^ uptake for the normal stimulation of insulin secretion demonstrated in the present and previous [3, 55] studies, it seemed important to understand whether and how the subtle fine tuning of cytosolic Ca^2+^ concentration by glucose and other secretagogues, including variations in oscillation frequency, regulates mitochondrial free [Ca^2+^] and consequently cytosolic ATP/ADP. Consistent with our recent findings [53], we show that in response to an elevation of glucose concentration from 3 to 16.7 mM, a progressive increases in [Ca^2+^]_mit_ occurs in the dissociated mouse β cells (Fig. 2a). This increase, which clearly lagged behind the increase in [Ca^2+^]_cyt_, was somewhat slower in onset and more stable than increases reported in dispersed rat islets by Wiederkehr and colleagues [55]. Importantly, [Ca^2+^]_mit_ remained elevated whilst [Ca^2+^]_cyt_ remained at the peak in the same cells. Furthermore, the application of high extracellular [K^+^] to depolarise the membrane [55] has been shown to stimulate the activity of plasma membrane Na^+^–K^+^ ATPase and hence alter both cellular energetic and ion homeostasis [56]. We note that parallel measurements of [Ca^2+^] in each compartment were also not possible in the earlier study [55] where a mitochondrially targeted aequorin was used to measure [Ca^2+^]_mit_ through bioluminescence recordings.

Using the more sensitive, fluorescence-based, probe for mitochondrial Ca^2+^, 2mt8RP [18] in single primary β cells, we show firstly that brief [Ca^2+^]_cyt_ spikes following a single (or a small number of) action potentials barely affect [Ca^2+^]_mit_ (Fig. 2a). However, the limited increases in mitochondrial Ca^2+^ that do occur under these conditions appeared to reverse slowly, such that a slow but progressive increase in [Ca^2+^]_mit_ could be observed. This integration or “summation” of the cytosolic Ca^2+^ peaks led us to explore the possibility that their frequency may control the eventual *amplitude* of the [Ca^2+^]_mit_ increases. By imposing such pulsatile changes in cytosolic Ca^2+^ through the manipulation of the membrane potential of single cells (Figs. 3 and 4), we provide evidence for such a model.

Thus, we show that β cell mitochondria achieve decoding of “frequency-tuned” cytosolic signals. What may be the physiological significance of this relationship? Firstly, we suspect that it provides a mechanism to ensure that the energetic demands of increased electrical activity are matched by elevated ATP synthesis, hence preventing the termination of the glucose signal (due to the re-opening of K_ATP_ channels). Secondly, it may mitigate the longer-term effects of supraphysiological stimulation, e.g. with sulphonylureas, a process akin to the excitotoxicity which leads to excessive Ca^2+^ influx and eventual cell death in neurons [27]. Thirdly, a positive feedback effect of enhanced electrical activity to increase ATP production may conceivably contribute to the steep, “switch-like” dose response of insulin secretion to glucose [31]. However, the activation of mitochondrial ATP synthesis by Ca^2+^ is likely to saturate and reverse above a certain limit (due to excessive Ca^2+^ accumulation, mitochondrial uncoupling and possibly the opening of permeability transition pores) [22]. The cell will then “reset” (thanks to the reopening of K_ATP_ and other channels) to a lower level of electrical and secretory activity.

Earlier evidence for the regulation of mitochondrial oxidative metabolism by cytosolic Ca^2+^ oscillations was obtained some years ago in hepatocytes [21], where mitochondrial pyridine nucleotide (NAD(P)H) fluorescence was assessed in response to vasopressin or other inositol 1,4,5-*tris*phosphate (IP_3_)-generating hormones. However, measurements of intramitochondrial Ca^2+^ were not performed in the previous study. Likewise, Pralong and colleagues [39] were also able to demonstrate in a variety of cell types (rat pancreatic β-, adrenal glomerulosa and liver) that oscillations in [Ca^2±^]_cyt_ were closely tracked by those in NAD(P)H fluorescence when the frequency of [Ca^2±^]_cyt_ oscillations remained low (for example, in β cells, at 8.3 mM glucose). By contrast, high-frequency cytosolic Ca^2±^ oscillations (e.g. at 11.2 mM glucose for β cells) induced the confluence of the individual NAD(P)H spikes and a stable elevation of this parameter. These earlier, and the present study, thus support the view that frequency of oscillations in cytosolic Ca^2+^ regulates the amplitude of the changes in fuel metabolism by mitochondria, adding to the list of cellular processes, such as gene expression in immune cells [16], which are controlled in this way. Such “demodulation” may therefore represent a common mechanism for the decoding by intracellular organelles of cytosolic Ca^2+^ signals, which avoids the potentially damaging consequences of more stable increases in [Ca^2+^]_cyt_.

### Roles for “mitochondrial plasticity” in the β cell?

An interesting observation made during the course of the present studies was the absence of any evident “desensitisation” of mitochondrial Ca^2+^ increases during repeated cytosolic Ca^2+^ pulses (Figs. S2 and 3), in contrast to previous findings in primary rat β- and insulinoma-derived INS1 cells [29] where a “run-down” in the increases was observed. Whether this reflects a difference in the behaviour of rat vs. mouse β cells, or the use of large populations of cells in the earlier study (where the behaviour of a cell sub-population may have biased recordings using aequorin), is presently unclear. On the other hand, and in contrast to the findings of Csordas and Hajnoczky in a mast cell line [9], we observed no increase in the amplitude of the successive [Ca^2+^]_mit_ increases when *V*_m_/[Ca^2+^]_cyt_ spikes were sufficiently well-spaced (e.g. at 0.5 min^−1^; Fig. S2) that an opportunity for mitochondrial Ca^2+^ accumulation between [Ca^2+^]_cyt_ peaks did not exist. Thus, Ca^2+^-dependent re-configuration of ER-mitochondria contacts (“mitochondrial plasticity”) [46] appears not be involved in regulating Ca^2+^ influx into β cell mitochondria.

By contrast, we provide further evidence (Fig. 6) that an alternative form of mitochondrial plasticity, described in earlier studies [1, 24], plays a role in shaping the metabolic responses of the β cell to glucose. Thus, after an initial drop, presumably reflecting increased cytosolic ATP consumption (e.g. for ion pumping), a steady and ultimately quite dramatic rise in ATP/ADP was observed, likely reflecting the stimulation of the intramitochondrial dehydrogenases and components of the respiratory chain [10].

Finally, the present studies also demonstrate that changes in ER Ca^2+^ are more sensitive to [Ca^2+^]_cyt_ increases than those in the mitochondria (Fig. 5). Thus, it appears that these two organellar systems (ER vs. mitochondria) may be able, at least in the β cell, to sense and decode [Ca^2+^]_cyt_ oscillations differently, a reflection of the distinct Ca^2+^ transporting machinery with which each is equipped. The consequences for ER function, notably protein synthesis, and for ER stress, are presently unclear.

## Conclusions

Extending our earlier findings [51, 53], we demonstrate here that (1) mitochondrial Ca^2+^ accumulation, mediated by MCU, is an important determinant of tolbutamide- and to a lesser extent glucose-stimulated insulin secretion from primary β cells and (2) that mitochondria in these cells integrate cytosolic pulses to modulate [Ca^2+^]_mit_ changes and ATP synthesis. Given the impairment in the normal pulsatility in insulin secretion observed in type 2 diabetes [34], it is conceivable that a derangement in the generation of cytosolic Ca^2+^ oscillations may even be a primary event which contributes to downstream production of ATP and hence insulin secretion. Whether pharmacological modulation of mitochondrial Ca^2+^ uptake may, therefore, regulate insulin secretion in vivo, and might thus provide a new approach to improve glucose tolerance in some forms of diabetes mellitus, is an intriguing question for the future.

## Electronic supplementary material

Below is the link to the electronic supplementary material.

Figure S1 **A** The standard voltage-clamp protocol used in this study. The protocol was designed to mimic natural-occurring electrical activity in β cells within intact islets. **B** Effect of high glucose (16.7 mM) measured in current clamp compared to the effect of depolarisation (2 min^−1^) applied to the same β cell in voltage clamp. The switch from current clamp to voltage clamp is indicated by the *arrow*. *V*_m_ was measured/manipulated using perforated patch configuration; [Ca^2+^]_cyt_ and [Ca^2+^]_mit_ were measured with Fura-Red and 2mt8RP, respectively. The trace is a representative of *n* = 10 cells. (JPEG 66 kb) (JPEG 58 kb)

High resolution image (TIFF 1728 kb)

Figure S2 Effect of extracellular glucose on free Ca^2+^ changes in the cytosol and mitochondria. **A** A single β cell was bathed in EC containing 10 mM glucose, with *V*_m_ being held at −70 mV. The depolarisations were then imposed using the patch pipette, with different frequencies being applied in random order. Glucose in the bath was then changed to 16.7 mM, as indicated, and the depolarisations were applied again. **B** Effect of the burst frequency on the [Ca^2+^]_cyt_ (*solid red*) and [Ca^2+^]_mit_ (*solid blue*), measured in 10 mM (*dashed line*, *circles*, *n* = 6) and 16.7 mM glucose (*solid lines*, *squares*, *n* = 13, equivalent to Fig. 2c). Maximal values of [Ca^2+^]_cyt_ and [Ca^2+^]_mit_ of each five-burst train were normalized to the maximal values of the trains with maximal frequency (10 min^−1^), measured in the same cell at the same glucose concentration. (JPEG 59 kb) (JPEG 78 kb)

High resolution image (TIFF 1830 kb)

Figure S3 Subcellular localisation of D4ER. Epifluorescence images of a cluster of three cells expressing D4-ER (2 days post infection), pre-incubated in Indo-1 (30 min). (JPEG 58 kb) (JPEG 19 kb)

High resolution image (TIFF 3071 kb)

Figure S4 Inhibition of sarco(endo)plasmidic reticulum Ca^2+^-ATPase (SERCA) does not cancel the “summation” of cytosolic Ca^2+^ increases by mitochondria. **A** Cyclopiazonic acid (CPA) inhibits the sequestration of Ca^2+^ into ER induced by depolarization. Data are representative of *n* = 5 traces. Cells were bathed in 16.7 mM glucose, and depolarisations were imposed using the patch pipette. [Ca^2+^]_ER_ (*green trace*) and [Ca^2+^]_cyt_ (*red trace*) were reported by D4-ER and Indo-1, respectively. **B** Effect of CPA on the depolarisation-induced dynamics of [Ca^2+^]_cyt_ and [Ca^2+^]_mit_. Data are representative of *n* = 5 traces. The cell was bathed in the EC solution containing 16.7 mM glucose, and [Ca^2+^]_cyt_ (*red trace*), [Ca^2+^]_mit_ (*blue trace*), and *V*_m_ (*upper trace*) were monitored/manipulated simultaneously. The cell was voltage-clamped at *V*_m_ = −70 mV, and the depolarisation protocol (Suppl. Fig. S1) was applied at the frequency of 2 and 4 min^−1^, as indicated. (JPEG 78 kb) (JPEG 66 kb)

High resolution image (TIFF 1776 kb)

Figure S5 Apparent Ca^2+^ entry into β cell mitochondria is favoured during influx from the extracellular space compared to mobilisation from intracellular stores. **A** Increases in [Ca^2+^]_cyt_ and [Ca^2+^]_mit_ induced by depolarization with KCl (*upper*) and application of acetylcholine (Ach; *lower*). **B** The inter-dependence between [Ca^2+^]_cyt_ and [Ca^2+^]_mit_ increases induced from extracellular solution, by depolarisation with 7.5, 10, 12, 15, 20 and 30 mM KCl (*red circles*), and from the intracellular stores, mobilised by 10^−3^, 10^−2^, 0.05, 0.08, 0.1, 1 and 100 μM acetylcholine (*green circles*). (JPEG 19 kb) (JPEG 59 kb)

High resolution image (TIFF 1695 kb)

## Abbreviations

[ATP/ADP]_cyt_: Apparent free cytosolic ATP/ADP ratio
[Ca^2+^]_cyt,_ [Ca^2+^]_mit_, [Ca^2+^]_ER_ cytosolic: Mitochondrial and endoplasmic reticulum free Ca^2+^ concentrations, respectively
ER: Endoplasmic reticulum
FCCP: Carbonyl cyanide 4-(trifluoromethoxy)phenylhydrazone
*G*_m_: Whole-cell conductance
*I*_m_: Whole-cell current
IP3: Inositol 1,4,5-*tris*phosphate
K_ATP_: ATP-sensitive K^+^ channels
MCU: Mitochondrial calcium uniporter
NCLX: Sodium-calcium (lithium) exchanger
ROI: Region of interest
T2D: Type 2 diabetes
*V*_m_: Plasma membrane electrical potential
