# Supplementary figures and images for: Frequency-dependent mitochondrial Ca2+ accumulation regulates ATP synthesis in pancreatic β cells
Source: Pflugers Arch. 2012 Nov 14;465(4):543–54. doi: 10.1007/s00424-012-1177-9 (PMC3631125; doi:10.1007/s00424-012-1177-9)

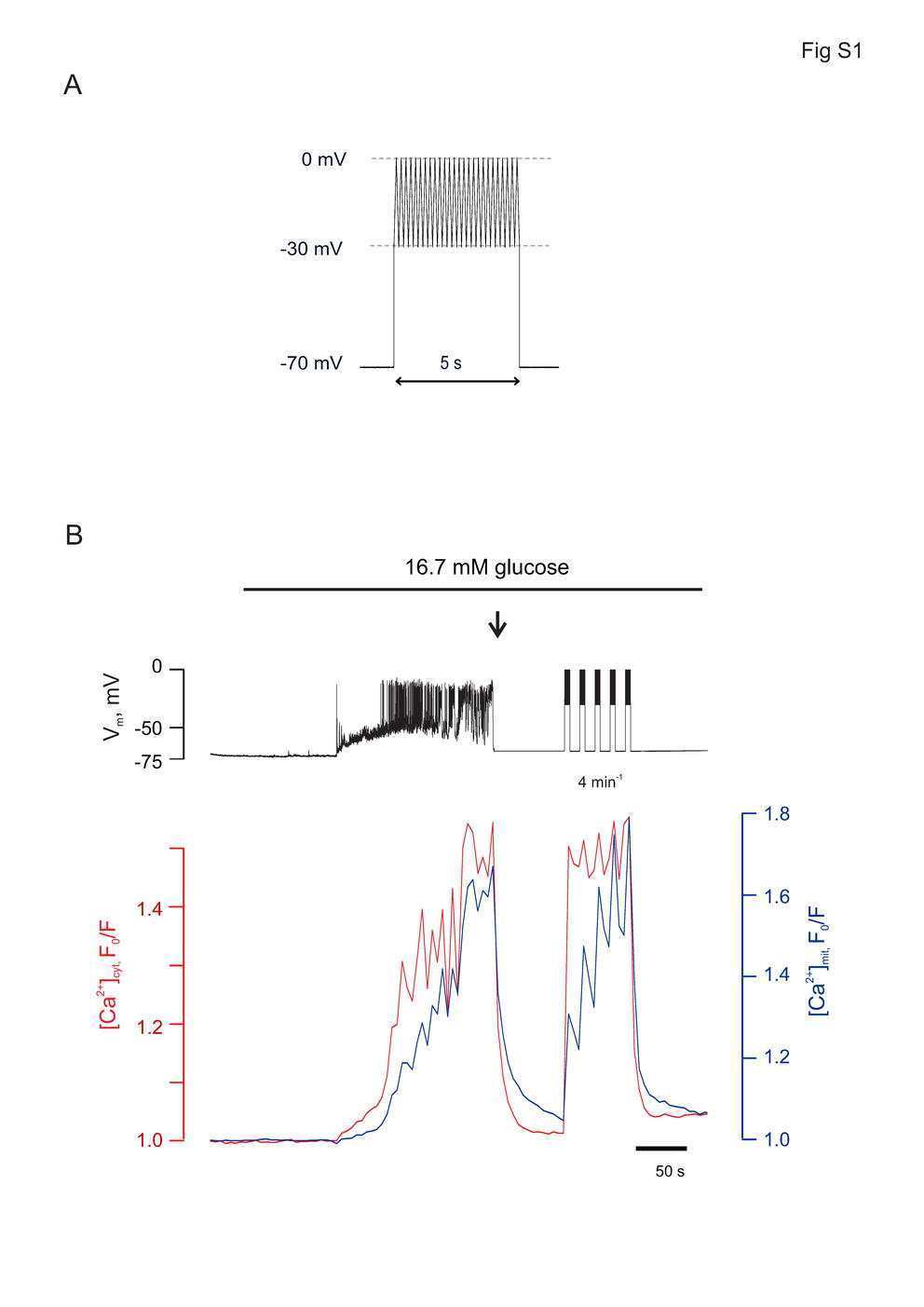

Supplement: Supplementary file 1 — A The standard voltage-clamp protocol used in this study. The protocol was designed to mimic natural-occurring electrical activity in β cells within intact islets. B Effect of high glucose (16.7 mM) measured in current clamp compared to the effect of depolarisation (2 min−1) applied to the same β cell in voltage clamp. The switch from current clamp to voltage clamp is indicated by the arrow. V m was measured/manipulated using perforated patch configuration; [Ca2+]cyt and [Ca2+]mit were measured with Fura-Red and 2mt8RP, respectively. The trace is a representative of n = 10 cells. (JPEG 66 kb) (JPEG 58 kb) [file 424_2012_1177_Fig7_ESM.jpg]

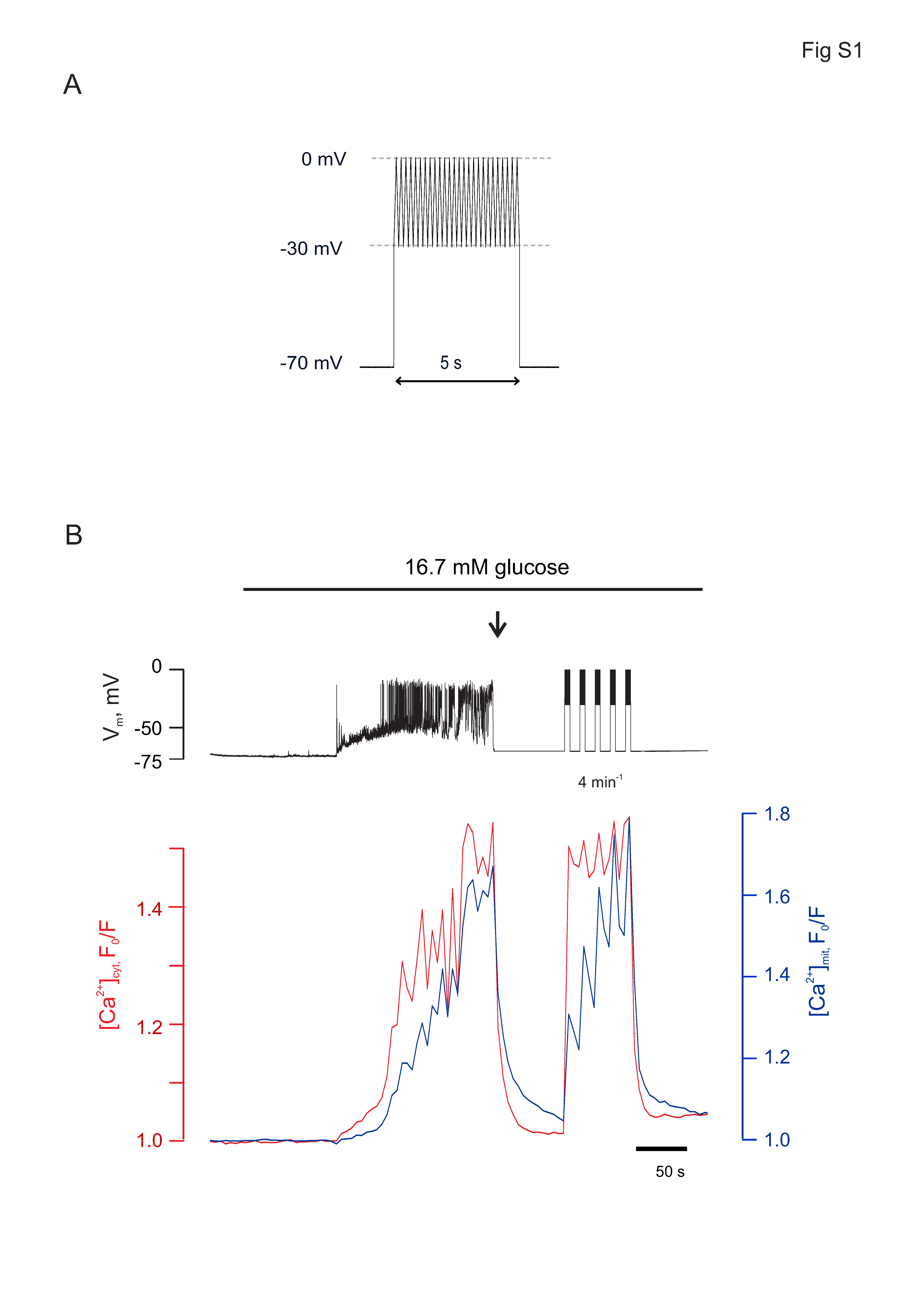

Supplement: Supplementary file 2 — High resolution image (TIFF 1728 kb) [file 424_2012_1177_MOESM1_ESM.tif]

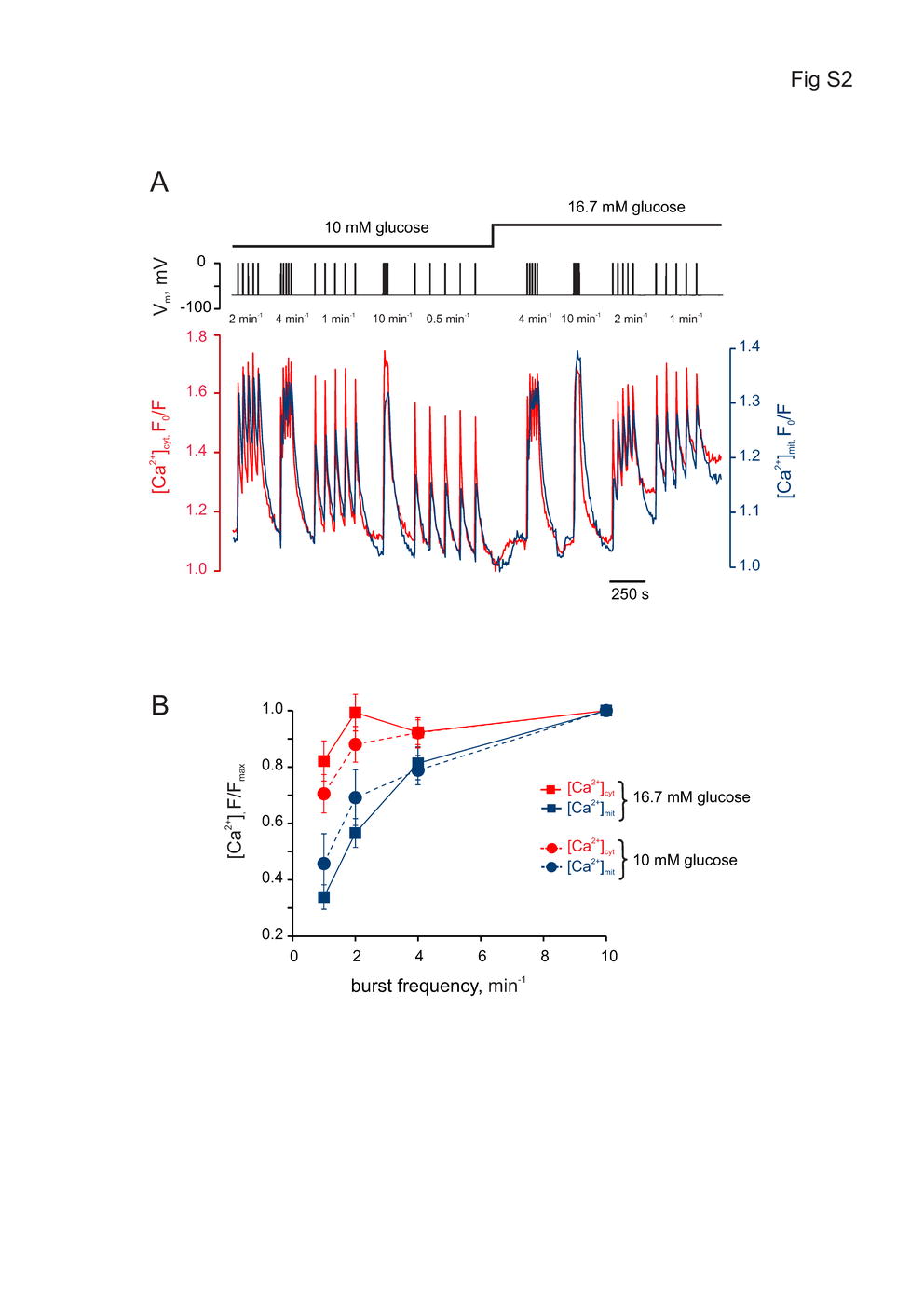

Supplement: Supplementary file 3 — Effect of extracellular glucose on free Ca2+ changes in the cytosol and mitochondria. A A single β cell was bathed in EC containing 10 mM glucose, with V m being held at −70 mV. The depolarisations were then imposed using the patch pipette, with different frequencies being applied in random order. Glucose in the bath was then changed to 16.7 mM, as indicated, and the depolarisations were applied again. B Effect of the burst frequency on the [Ca2+]cyt (solid red) and [Ca2+]mit (solid blue), measured in 10 mM (dashed line, circles, n = 6) and 16.7 mM glucose (solid lines, squares, n = 13, equivalent to Fig. 2c). Maximal values of [Ca2+]cyt and [Ca2+]mit of each five-burst train were normalized to the maximal values of the trains with maximal frequency (10 min−1), measured in the same cell at the same glucose concentration. (JPEG 59 kb) (JPEG 78 kb) [file 424_2012_1177_Fig8_ESM.jpg]

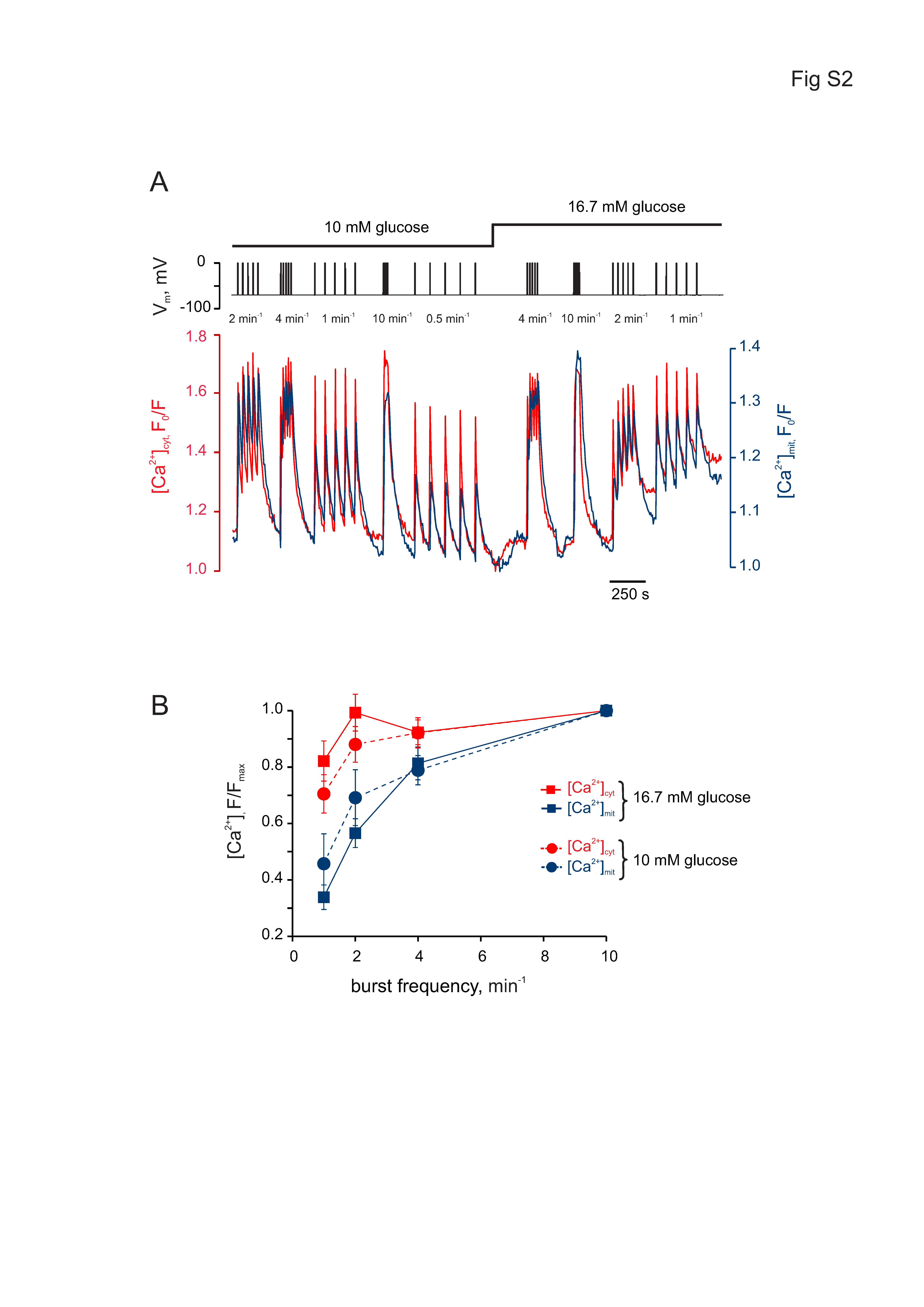

Supplement: Supplementary file 4 — High resolution image (TIFF 1830 kb) [file 424_2012_1177_MOESM2_ESM.tif]

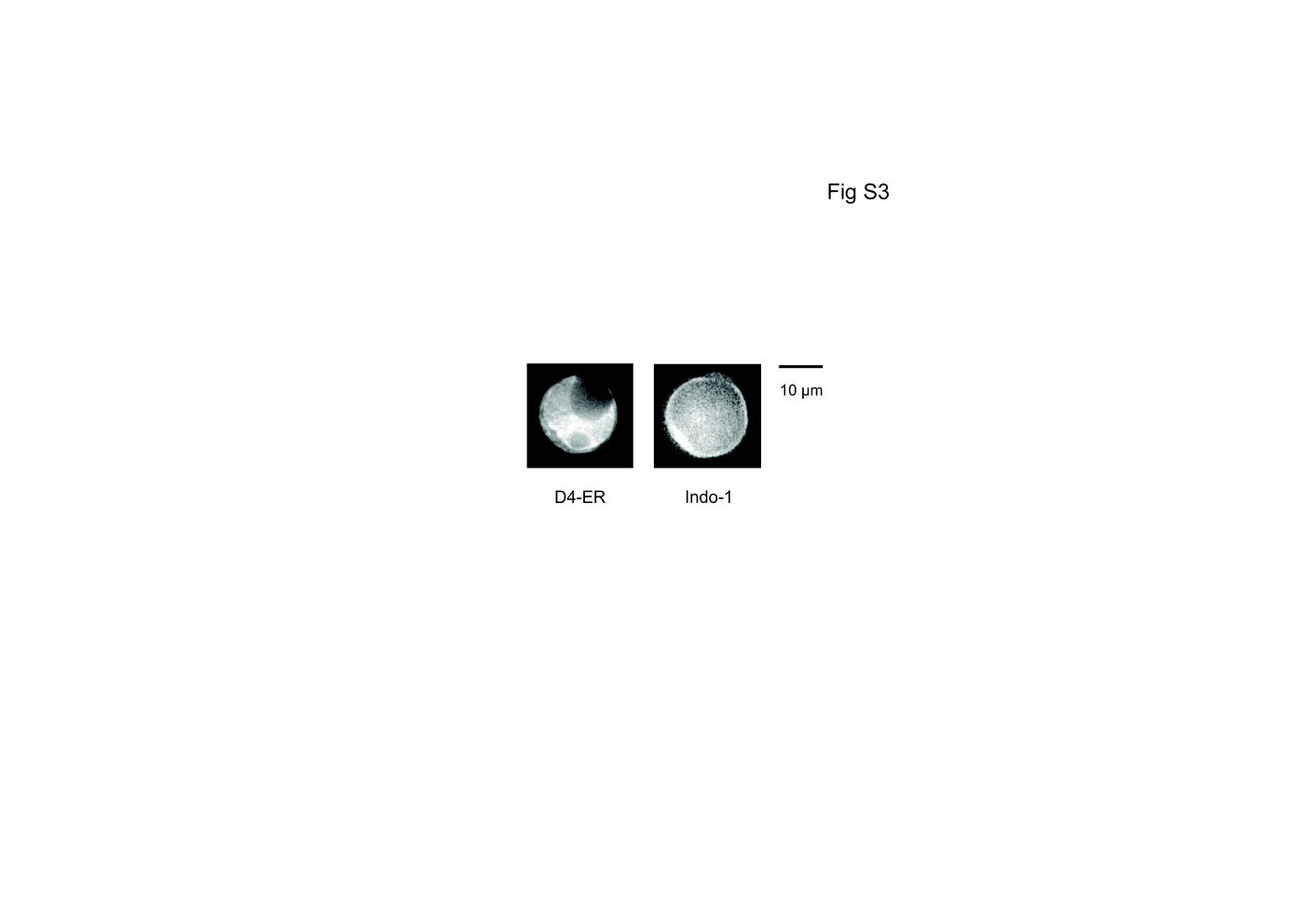

Supplement: Supplementary file 5 — Subcellular localisation of D4ER. Epifluorescence images of a cluster of three cells expressing D4-ER (2 days post infection), pre-incubated in Indo-1 (30 min). (JPEG 58 kb) (JPEG 19 kb) [file 424_2012_1177_Fig9_ESM.jpg]

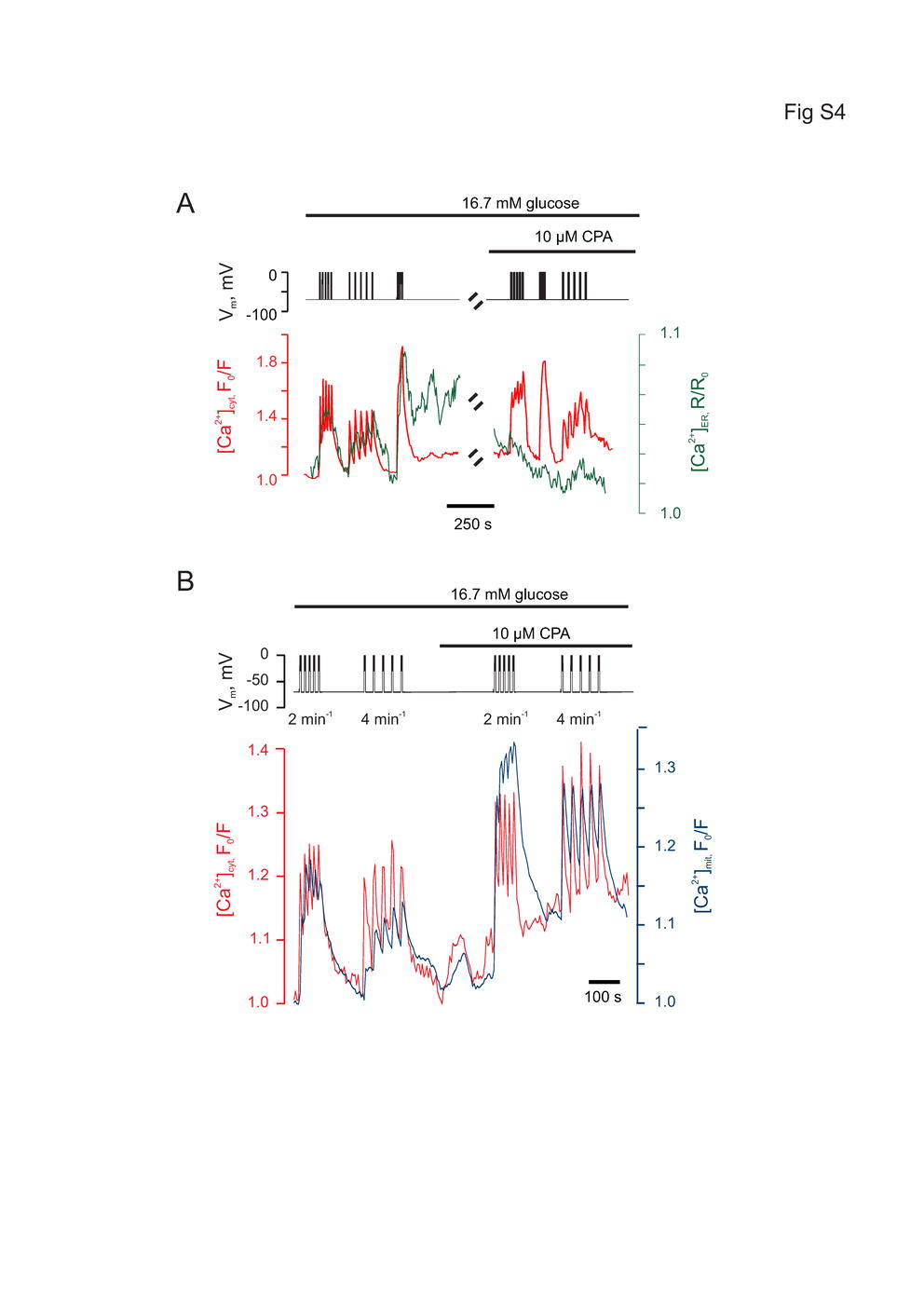

Supplement: Supplementary file 7 — Inhibition of sarco(endo)plasmidic reticulum Ca2+-ATPase (SERCA) does not cancel the “summation” of cytosolic Ca2+ increases by mitochondria. A Cyclopiazonic acid (CPA) inhibits the sequestration of Ca2+ into ER induced by depolarization. Data are representative of n = 5 traces. Cells were bathed in 16.7 mM glucose, and depolarisations were imposed using the patch pipette. [Ca2+]ER (green trace) and [Ca2+]cyt (red trace) were reported by D4-ER and Indo-1, respectively. B Effect of CPA on the depolarisation-induced dynamics of [Ca2+]cyt and [Ca2+]mit. Data are representative of n = 5 traces. The cell was bathed in the EC solution containing 16.7 mM glucose, and [Ca2+]cyt (red trace), [Ca2+]mit (blue trace), and V m (upper trace) were monitored/manipulated simultaneously. The cell was voltage-clamped at V m = −70 mV, and the depolarisation protocol (Suppl. Fig. S1) was applied at the frequency of 2 and 4 min−1, as indicated. (JPEG 78 kb) (JPEG 66 kb) [file 424_2012_1177_Fig10_ESM.jpg]

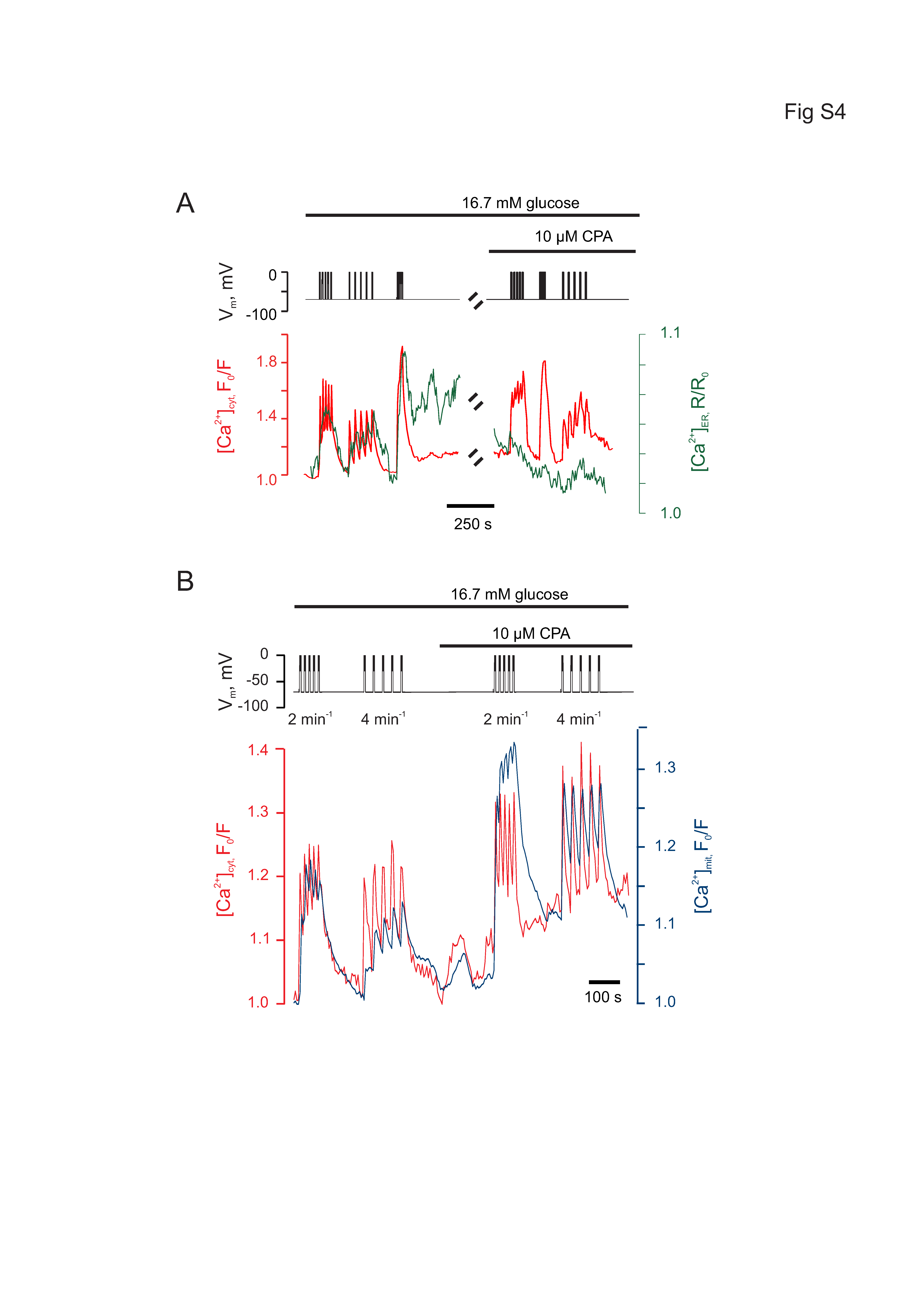

Supplement: Supplementary file 8 — High resolution image (TIFF 1776 kb) [file 424_2012_1177_MOESM4_ESM.tif]

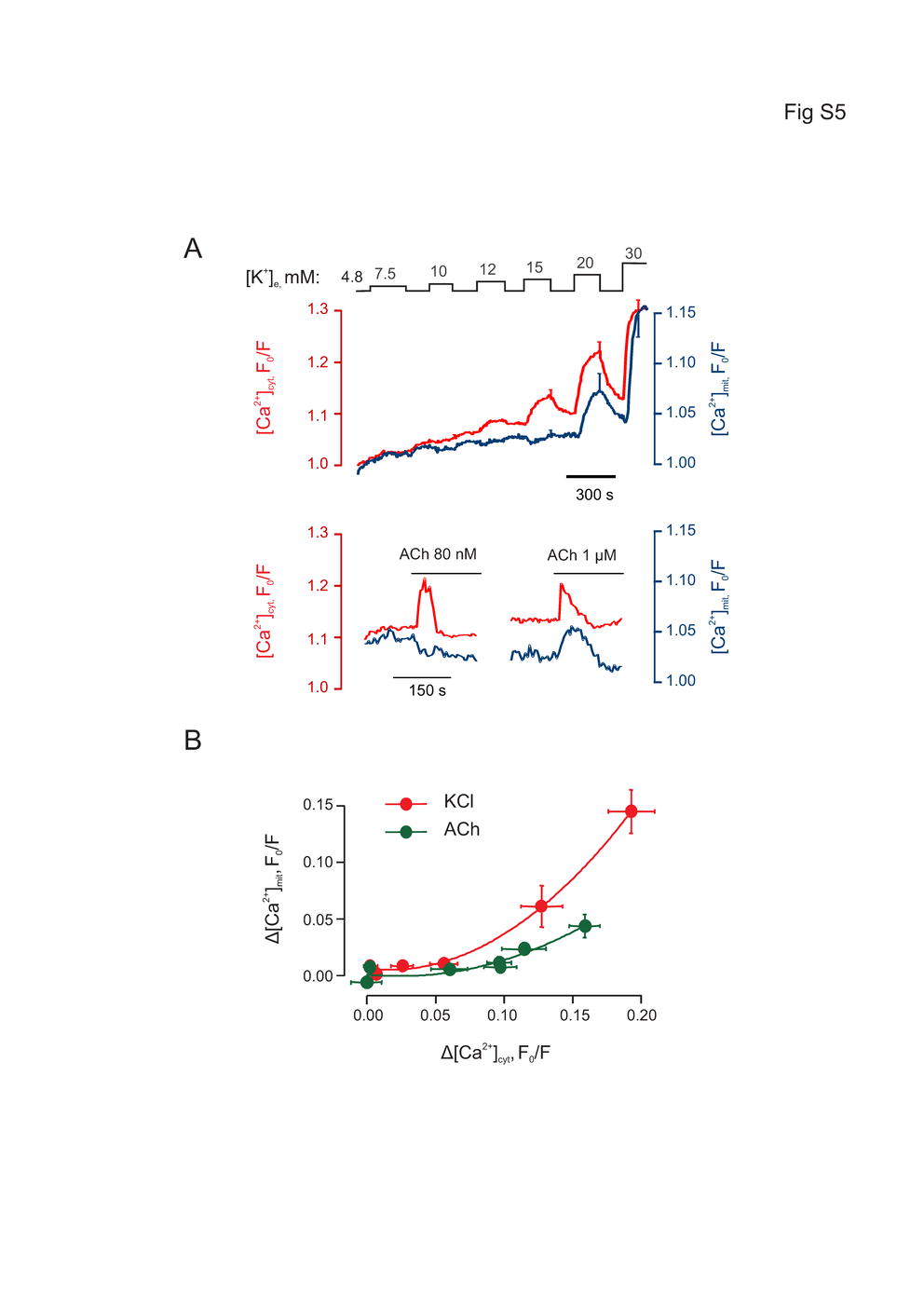

Supplement: Supplementary file 9 — Apparent Ca2+ entry into β cell mitochondria is favoured during influx from the extracellular space compared to mobilisation from intracellular stores. A Increases in [Ca2+]cyt and [Ca2+]mit induced by depolarization with KCl (upper) and application of acetylcholine (Ach; lower). B The inter-dependence between [Ca2+]cyt and [Ca2+]mit increases induced from extracellular solution, by depolarisation with 7.5, 10, 12, 15, 20 and 30 mM KCl (red circles), and from the intracellular stores, mobilised by 10−3, 10−2, 0.05, 0.08, 0.1, 1 and 100 μM acetylcholine (green circles). (JPEG 19 kb) (JPEG 59 kb) [file 424_2012_1177_Fig11_ESM.jpg]

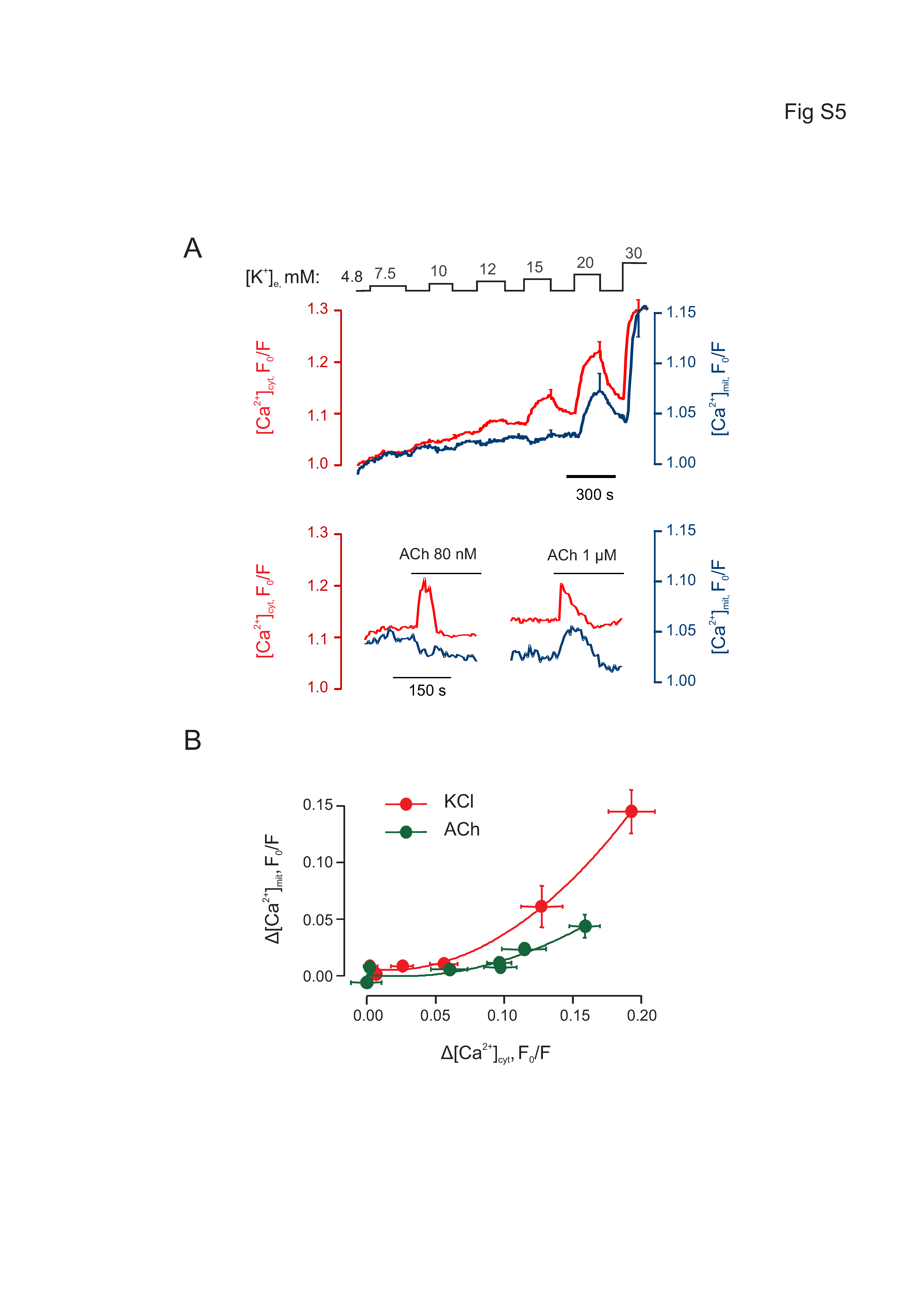

Supplement: Supplementary file 10 — High resolution image (TIFF 1695 kb) [file 424_2012_1177_MOESM5_ESM.tif]
